# Oral submucous fibrosis: pathogenesis and therapeutic approaches

**DOI:** 10.1038/s41368-024-00344-6

**Published:** 2025-02-01

**Authors:** Jianfei Tang, Junjie Liu, Zekun Zhou, Xinyan Cui, Hua Tu, Jia Jia, Baike Chen, Xiaohan Dai, Ousheng Liu

**Affiliations:** Hunan Key Laboratory of Oral Health Research & Hunan 3D Printing Engineering Research Center of Oral Care & Hunan Clinical Research Center of Oral Major Diseases and Oral Health & Academician Workstation for Oral-Maxilofacial and Regenerative Medicine & Xiangya Stomatological Hospital & Xiangya School of Stomatology, Central South University, Changsha, China

**Keywords:** Oral diseases, Mechanisms of disease

## Abstract

Oral submucous fibrosis (OSF), characterized by excessive deposition of extracellular matrix (ECM) that causes oral mucosal tissue sclerosis, and even cancer transformation, is a chronic, progressive fibrosis disease. However, despite some advancements in recent years, no targeted antifibrotic strategies for OSF have been approved; likely because the complicated mechanisms that initiate and drive fibrosis remain to be determined. In this review, we briefly introduce the epidemiology and etiology of OSF. Then, we highlight how cell-intrinsic changes in significant structural cells can drive fibrotic response by regulating biological behaviors, secretion function, and activation of ECM-producing myofibroblasts. In addition, we also discuss the role of innate and adaptive immune cells and how they contribute to the pathogenesis of OSF. Finally, we summarize strategies to interrupt key mechanisms that cause OSF, including modulation of the ECM, inhibition of inflammation, improvement of vascular disturbance. This review will provide potential routes for developing novel anti-OSF therapeutics.

## Introduction

Oral submucous fibrosis (OSF) is a chronic, progressive disease of the oral mucosa that results in fibrosis and leads to limitation in mouth opening, burning sensation, xerostomia, dysphagia, and even facial deformity, which seriously influences patients’ quality of life. It is worth noting that OSF has a potential tendency for malignant transformation. The World Health Organization (WHO) has listed OSF as one of the oral potentially malignant disorders (OPMDs) and ~3%–10% of patients with OSF may eventually develop oral cancer.^1,2^ However, despite substantial progress in the understanding of the pathobiology of various organ fibrosis, the investigation of OSF was largely deficient due to the lack of attention to this disease and the limitations in the field of research. What is consistent with other organ fibrosis diseases is that huge obstacles remain between the identification of ideal antifibrotic strategies and the implementation of this concept into clinical application.

**Fig. 1:**
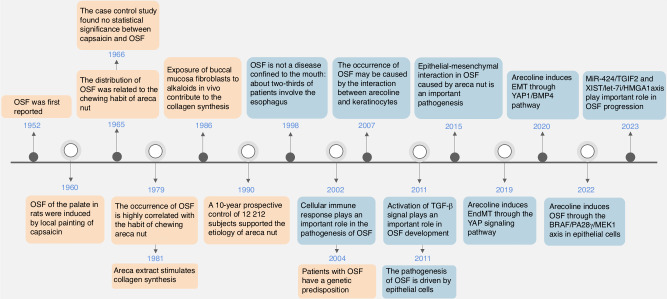
Timeline for key events in studies of OSF research. Orange boxes summarize etiological studies of OSF; Blue boxes show relevant studies of OSF pathogenesis

Currently, the exploration of OSF has been carried out for half a century and has been mainly focused on etiology and pathogenesis^3–21^ (Fig. 1). Relative studies of patients with OSF along with experimental models of OSF in rodents have preliminarily demonstrated potential mechanisms leading to OSF. These include activation of fibroblast, damage to the epithelial barrier, injury of endothelial cell, dysregulation of immune responses, microvascular lesions, and the roles of oxidative stress, senescence, and autophagy. However, the underlying molecular mechanisms remain controversial. In general, strategies for individual and precise therapy of OSF are the ultimate goals to be explored. This review summarizes mechanisms and antifibrotic strategies outlined in the existing studies. The improvement in comprehending possible mechanisms including the role of oral microbiota, immune regulation, and the promising innovations will be discussed to introduce novel perspectives for the therapy of OSF.

## Epidemiology and Etiology

According to epidemiological statistics, there are about 600 million people with the habit of chewing areca nut (AN) in the world, and ~5% of AN chewers are OSF patients.^22,23^ Numerous studies from different countries supported the correlation between AN chewing and OSF.^24–26^ It is reported that the prevalence rate of OSF in China is above 1.0%.^27^ The prevalence rate of other south-east Asia countries such as India and Vietnam is 0.04%–6.4% and 0.15%–14.6%, respectively.^26,28^ The prevalence across these countries is greatly attributed to the habit of chewing AN. Most strikingly, the rate of transformation into oral cancer in OSF patients is above 4%.^29,30^ A prospective study over 30 years in mainland of China showed that 32 of 567 (5.6%) OSF patients with habits of chewing AN transformed into oral cancer.^31^ In Mumbai and Lucknow, above 2.0% and 9.8% of patients with oral squamous cell carcinoma (OSCC), respectively, endured malignant transformation from OSF.^32^ Another follow-up study of 17 years revealed that 5 of 66 (7.6%) Indian OSF patients had malignant transformation into OSCC.^33^ These studies support the hypothesis that OSF has malignant transformation potential and suggest that OSF is one of the most important public health problems. However, the current viewpoints of etiology in OSF are multifactorial. The chemical and physical irritation, genetic factors, as well as metabolic disturbance, are acknowledged factors in the onset and development of OSF.

### The chemical and physical irritation

The chemical and physical irritation caused by AN is often considered the most significant risk factors in OSF onset. Specifically, the International Agency for Research on Cancer (IARC) has assembled evidence presenting that chemical and physical irritation by chewing AN is the major cause of OSF in their monograph.^34^ Many studies suggested that the composition of AN can lead to persistent chemical trauma in the oral mucosa. Until now, the identified chemical composition of AN is comprised of alkaloids (such as arecoline, arecaidine, guvacine, and guvacoline), flavonoids, tannins, along with carbohydrates, proteins, fats, crude fiber, and other elements.^35^ In particular, the arecoline component is confirmed to be the most potent agent which impacts the occurrence of OSF by inducing abnormal deposition of collagen.^36^

Chronic mechanical irritation (CMI) is important in the onset and development oral mucosal lesions. Some pathological changes such as atrophy, ulceration, keratosis, hyperplasia, and even fibrosis will appear in the oral mucosa under direct contact with a ‘mechanical agent’ (e.g., teeth or denture) and CMI. As a result, OSF is also usually considered to be a result of mechanical trauma of the oral mucosa by the coarse fibers of AN. Pentenero et al. hold the idea that mechanical trauma is a common phenomenon which is caused by AN chewing, and it may initiate negative functions in DNA reparation and cell apoptosis.^37^ However, the mechanical irritation is a local promotive factor rather than a main direct factor. It has been reported that mechanical trauma by AN coarse fiber can aggravate OSF. Yang et al. found that rubbing arecoline in a particular area significantly promoted the expression of collagen III and TGF-β1 in SD rat model, compared with rubbing alone.^38^

### Genetic susceptibility

Previous studies also provided evidence of genetic susceptibility to OSF from the perspective of gene polymorphisms and cytogenetic damage. These genetic factors contribute to pathogenesis of OSF, especially the changes in gene polymorphisms, (e.g., The increased gene frequency of human leukocyte antigen (HLA), cytotoxic T lymphocyte-associated antigen-4 (CTLA-4), glutathione s-transferase (GST), cytochrome P450 (CYP) and matrix metalloproteinase (MMPs)) are main factors in genetic susceptibility to OSF.

A study reported that the frequency of HLA-B76 and the haplotype frequencies of HLA-B48/Cw7, -B51/Cw7, and -B62/Cw7 were significantly higher in OSF patients than in healthy controls. It also suggested that some specific HLA haplotypes may play a more important role in OSF susceptibility than individual HLA phenotypes.^39^ However, there was a contrary view from South Africa that OSF had no HLA-associated susceptibility.^40^ Furthermore, other studies have concluded that gene polymorphisms of MHC class I chain-associated gene A (MICA) and the CTLA-4 might confer an increased risk of OSF.^41–43^ Another comparative study of 90 patients with OSF and 130 healthy controls also reported the GST variants—GSTM1 and GSTT1, increase the risk of OSF progression in the North Indian population.^44^ Similarly, the expression of MMP-3,^45^ CYP3A,^46^ CYP1A1 and CYP2E1^47^ were also shown to have a significant role associated with OSF susceptibility.

In addition, some evidence showed that OSF patients have cytogenetic damage, manifested by elevated indicators such as sister chromatid exchange rates,^48,49^ micronucleus cell numbers^50,51^ and degree of DNA damage,^52^ which can be attributed to the genotoxic effects of AN chewing. These findings suggest that OSF has a genetic background and susceptibility, and that susceptible individuals may have unstable chromosomes with reduced repair capacity and are more susceptible to fibrosis when stimulated by external factors.

### Metabolic disturbance

Many studies have found a correlation between metabolic disturbance and susceptibility to OSF by comparing the differences in indicators of trace elements, vitamins, lipids, and proteins between normal subjects and OSF patients.

Many studies have detected trace element levels in OSF and have reported their association with OSF. A meta-analysis containing 34 reports showed that the levels of saliva, serum, or plasma copper were significantly elevated in OSF patients, whereas zinc and iron were significantly decreased.^53^ Of note, lysyl oxidase (LOX) is a copper-dependent monoamine oxidase that works in collagen cross-linking and deposition. Its activity was found to be upregulated in fibroblasts of OSF tissue.^54^ Based on this finding, one view suggested that excessive copper intake caused by AN chewing led to the upregulation of LOX activity and the occurrence of fibrosis.^55^ Furthermore, OSF patients had a significantly decreased lipid profile. One study showed that plasma total cholesterol (TC), high-density lipoprotein (HDL), and Apo-A1 levels were decreased,^56^ while another study showed that serum TC, HDL, and low-density lipoprotein (LDL) were decreased. Perhaps the lipid profile can be used as an indicator for detecting initial changes in cells of OSF.^57^ Most importantly, these factors are not isolated, but are interrelated and interact with each other.

## Pathogenesis of OSF

**Fig. 2:**
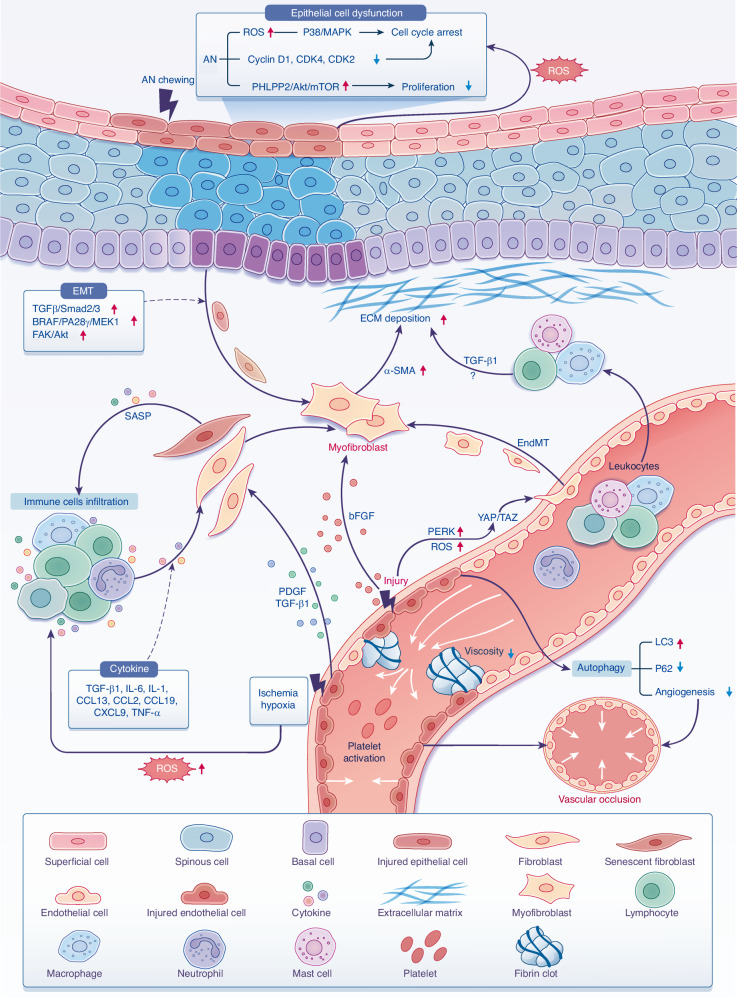
The pathogenesis of OSF. The damage of oral epithelial cells through the production of ROS or through the dysfunction of epithelial cells, such as arrested cell cycle and proliferation inhibition work to promote fibrosis. In addition, the injured epithelial cells also promote EMT process by activating EMT-related signaling pathways. The vascular endothelial injury caused by arecoline also leads to the production of ROS, thus promoting EndMT by activating YAP/TAZ and can eventually leads to fibrosis. Infiltrated immune cells secrete cytokines induced by injury, local ischemia, hypoxia, and SASPs, which were derived from fibroblasts in senescence conditions, thus promoting fibrosis. Leukocytes from circulation may be recruited by chemokines to inflamed areas to secrete profibrotic cytokines. Furthermore, secreted mediators such as TGF-β1, PDGF, bFGF cause fibroblasts activation, which produces excessive ECM. Injured endothelial cells also induce autophagy, fibrinolysis system disorder, and abnormal coagulation function, which leads to microvascular occlusion and further accelerates the process of fibrosis

OSF is considered to be an excessive repair process that occurs after persisting chronic injury. In this regard, almost all the cell types in the oral mucosa, including epithelial cells, fibroblasts, endothelial cells, as well as the infiltrated immune cells, are involved and participate in some way in the pathogenesis of OSF, which illustrates the immense complexity of this process. In this context, understanding the cellular and molecular mechanism of OSF is paramount and essential, including fibroblast activation, epithelial damage, submucosal vasculature abnormality, immune abnormality, as well as other mechanisms such as oxidative stress, autophagy, and senescence (Fig. 2). All of the above provide a novel insight into pathogenesis of the OSF process.

### Fibroblast activation

During the development of OSF, persistent and chronic tissue injury inevitably leads to the massive activation of fibroblast, which is arguably an important event in oral mucosa fibrogenesis. These activated fibroblasts are commonly regarded as the principal matrix-producing cells which have skeleton protein phenotype (such as α-SMA) and generate a large amount of mesenchymal matrix components, including fibronectin-1 (FN-1) and collagen I, also known as myofibroblast (MFB).^58^ MFBs were observed in oral mucosa with OSF patients, and the number of MFBs is significantly correlated with the severity of OSF.^59^ Further study indicated that buccal mucosa fibroblasts obtained from OSF patients show a significant upexpression of α-SMA, collagen I, FN-1, and collagen gel contraction compared to fibroblasts from healthy subjects.^60^ Notably, TGF-β is generally regarded as one of the most potent activators known to induce fibroblast activation.^61^ Canonical and non-canonical signaling pathways involving TGF-β often contribute to fibrosis pathogenesis. For example, the activation of TGF-β induces phosphorylation of Smad2 to promote fibroblast differentiation-specific gene expression in vitro.^62^ In addition to Smad signaling, TGF-β also activates fibroblasts by induction of non-canonical signaling such as JNK, and p38 MAPK pathways.^63,64^

Moreover, AN extract is an important pathogenic factor in OSF, and it exerts a crucial role in determining fibroblast activation. In vitro studies confirmed that AN extract markedly induces the high expression level of vimentin of fibroblasts, corresponding to the high expression of vimentin in the fibroblasts from OSF patients.^65^ Certainly, vimentin is a significant marker for fibroblast activation, as it is expressed de novo specifically in activated, but not quiescent, fibroblasts of adult kidneys.^66^ Cultured fibroblasts respond to AN extract or arecoline in vitro, and experience differentiation into cells that exhibit features of MFBs including α-SMA expression and collagen gel contraction, and other fibrosis marker expression, such as CTGF.^67–70^ In addition, buccal mucosa biopsies of subjects with OSF showed increase of SSEA-4 and ZEB-1, as well as non-coding RNAs (such as H19, linc000312 and HOTTIP). These genes were upregulated in fibroblasts treated with AN extract, and promoted the transformation of fibroblasts into MFBs.^60,67,71–73^ Further research found that AN extract induces the transformation of fibroblasts into MFBs through multiple molecular mechanisms, including activation of PLC/IP3/Ca2^+^ and Rho kinase regulating cell contraction, as well as direct phosphorylation by NF-κB, JNK, P38 MAPK mediating CTGF production.^68,69^ Of note, another mechanism that AN may indirectly influence is fibroblastic collagen metabolism by keratinocytes. For example, keratinocytes collected from healthy oral mucosa treated by AN extract induce TIMP-1 released by fibroblasts.^74^ ECM-related proteins released by fibroblasts have been described in other literature.^65,75–77^ In addition, AN also directly inhibits fibroblastic collagen degradation. Studies reported that AN caused a dose-dependent inhibition of collagen phagocytosis by fibroblast in vitro. Furthermore, a deficiency in collagen phagocytosis was noted in OSF fibroblasts relative to fibroblasts cultured from healthy buccal mucosa.^78^ However, more studies will need to be conducted to characterize their contribution to OSF.

### Epithelial damage

Epithelial cells derived from oral mucosa, which include basal cells, spinous cells, and superficial cells, are essential cells in maintaining the integrity of the epithelial wall and tissue homeostasis in oral submucosa.^79^ During the lesion of OSF development, the stimulation of chewing AN will result in the damage of epithelial cells, thus destroying epithelial structure, and promoting dysfunctional repair. A series of studies showed that AN extract has significantly toxic effect to the proliferation of keratinocytes and induces cell apoptosis in a concentration-dependent manner.^80,81^ In addition, the treatment of AN extract also caused the cell cycle of injured epithelial cells to be arrested at the G1/G0 phase and suppressed epithelial cell viability by activation of important signaling pathways.^82,83^ For example, the arecoline suppressed epithelial cell viability and proliferation by activating TGF-β/Smad or PHLPP2/Akt/mTOR signaling pathway to promote OSF development.^84,85^

Furthermore, AN can damage oral epithelial cells leading to epithelial-mesenchymal transition (EMT), which is considered to be an important process in the development of OSF. The EMT process is characterized by substantial change of normal epithelial structure, that epithelial cells lose contact and adhesion markers (such as E-cadherin), and obtain mesenchymal markers (such as α-SMA, vimentin, and N-cadherin) through drastic changes in the shape of cytoskeleton.^58^ These cell-intrinsic changes in epithelial cells have also been shown to promote the occurrence of OSF by maintaining the activation of some key profibrotic pathways.^86,87^ For instance, Xie et al. found that arecoline, as the pathogenic component of OSF, can upregulate the levels of PA28γ, BRAF, and phosphorylated MEK1 protein, which interact with each other to form a protein complex, thus activating MEK1/ERK signaling pathway and promoting EMT in oral epithelial cells.^19^ Other studies also revealed that over-expression of DEC1 in the oral epithelium of OSF mice, along with synchronized activation of FAK/Akt signaling pathways, which caused EMT in epithelial cells, and promoted fibrosis development.^88^

In addition, damaged epithelial cells secrete high levels of cytokines, such as TGF-β, which stimulates the proliferation of fibroblasts and increases the deposition of ECM, driving the development of OSF.^16^ The expression levels of TGF-β are elevated in the oral epithelium, up to the stratum spinosum from OSF patient tissue.^89^ Concurrently, the TGF-β signaling pathway is the main cascade reaction of fibroblast differentiation. A recent study demonstrated that arecoline enhanced the secretion of TGF-β1 by epithelial cells, which promoted the expression of THBS1 in fibroblasts, thereby contributing to OSF development.^90^ Notably, Pant et al. reported that the OSF invoking involvement of JNK/ATF2/Jun axis in TGF-β/Smad pathway activation by AN extract treatment leads to a stained TGF-β autocrine loop in epithelial cells which promotes OSF.^63^

Hence, epithelial damage has an important role through EMT or cooperation of TGF-β related signaling pathways in the initiation and development of the OSF. However, the mechanisms associated with EMT into tissue fibrosis have not been fully identified. Exactly what triggers the formation of the EMT after epithelial injury is an unresolved question. How the EMT cells are initially migrated to mesenchymal area is unknown. Future exploration of the pro-fibrotic role of epithelium should provide further insight into the pathogenesis of OSF.

### Submucosal vasculature abnormality

Functional vasculature is significant in the process of tissue repair and fibrosis, and it is essential for maintaining the stability of oral mucosa by transporting oxygen and other nutrients. Previously, observations of OSF reported abnormal vascular changes, including incomplete vascular basement membrane, low microvascular density, and diameter around areas of fibrosis.^91^ More detailed study of 40 cases of OSF patients later clarified that with the development of OSF, the number of blood vessels gradually narrows and decreases, even completely occluded in the advanced stage, resulting atrophic changes and subsequent fibrosis.^92,93^ In addition, AN extract has a cytotoxic effect on endothelial cells, and a study has reported that arecoline markedly inhibits the proliferation of vascular endothelial cells, promotes apoptosis, and induces the arrest of cell cycle, thus causing vascular damage and dysfunction and leading to fibrosis.^94^ However, it is not clear how these vascular abnormalities cause OSF. Several mechanisms have been proposed for abnormal vascular patterns in OSF.

First, the injured endothelial cells are confirmed to promote inflammation and indirectly activate MFB and ECM deposition. Li et al. found high expression of CXCL9 in the endothelial cells and immune cells by observing 56 OSF patients with AN chewing habit.^95^ It suggests that AN extract changes the adhesive profile of endothelial cells by increasing the expression of the surface proteins that promote the attraction of circulating leukocytes to it, thus, facilitating the initiation or maintenance of vascular inflammation. Other studies revealed that exposing human umbilical vein endothelial cells (HUVECs) to arecoline increases the expression of bFGF and ET-1 while down-regulating interleukin (IL)-1, IL-6, G-CSF, and GM-CSF, and these abnormally secreted cytokines by endothelial cells further regulate the proliferation of fibroblasts and promote the occurrence and development of OSF.^96^

Secondly, coagulation and fibrinolysis systems also could be modulated by injured endothelial cells, thus promoting the development of OSF. A previous study by Phatak reported that the blood of patients with OSF was hypercoagulable and accompanied by fibrinolysis disorder, which suggested that OSF is a chronic disseminated intravascular coagulation syndrome (DIC) with local coagulopathy.^97^ Fang et al. reported that there was an extensive microvascular injury in OSF, accompanied by vasodilation and increased permeability.^98^ The plasma leakage and fibrin deposition caused by these pathological changes further activated the coagulation mechanism, which led to the decrease of blood flow and fibrosis in oral mucosa tissue. Moreover, arecoline can effectively stimulate platelet aggregation and thromboxane production, causing the activation of local blood coagulation mechanism. Another study also demonstrated the fibrosis level was regulated by high expression of PAI-1, a plasminogen inhibitor, which will lead to the decline of plasminogen activity, as well as the degradation of ECM.^99^

Thirdly, injured endothelial cells may transdifferentiate towards MFBs, which contributes to OSF. A typical example is that AN induces endothelial cell to undergo endothelial‐to‐mesenchymal transition (EndMT) in vitro. In this study. the activation of PERK pathway causes the expression of transcription factors such as Yes-associated protein (YAP), which subsequently initiates EndMT and promotes OSF development.^17^ Overexpression of nuclear receptor coactivator 7 (NCOA7) reduced AN-induced EndMT. Furthermore, the specific YAP inhibitor, Verteporfin, has also been found to reduce collagen accumulation through inhibition of EndMT, thereby alleviating OSF in mice.^100^ Comprehensive studies are required and understanding of EndMT processes is pivotal for OSF treatment.

### Immune abnormality

Immune abnormalities, including dysregulated immune cells and overproduction of pro-inflammatory or pro-fibrotic molecules, are one of the main features of OSF pathogenesis, which is involved in OSF development.^101^ Several studies have reported that monocytes/macrophages, mast cells (MCs), and T cells expand in OSF, with increased expression of cytokines, including CCL19, IL-1α, IL-1β, IL-6, TNF-α, and TGF-β1 in tissue and peripheral blood mononuclear cells (PBMCs) of patients with OSF. Both the innate and adaptive immune responses have critical roles in the fibrotic process and promote disease development in patients with OSF.

In the innate immune response, macrophages are commonly regarded as the key immune regulator, which participates in various organ fibrosis. Macrophages have a high plasticity, which results in a broad spectrum of macrophage phenotypes. The monocytes that later transform into inflammatory macrophages seem to be the main immune cells in fibrosis formation.^102^ Numerous studies have shown that targeted deletion of monocyte-derived macrophages (CCR2^+^ macrophage) is effective in alleviating fibrosis of lung, liver, and kidney.^103–105^ Notably, a previous immunohistochemical study showed that the infiltration of CD68^+^ macrophages in OSF tissue increased, and the level of infiltration was positively correlated with the aggravation of pathological stage.^106^ Interestingly, the onset of OSF accompanied by the upregulation of CCR2 and CCL2; this phenomenon seems to support the consistent profibrotic role of monocytes/CCR2^+^ macrophage in OSF as previously reported in other organ fibroses.^107–109^ However, the specific phenotypes of macrophages in OSF are unproven and need comprehensive investigation. Apart from macrophages, MCs and natural killer (NK) cells are also emerging as innate immune effectors with central roles in the pathogenesis of OSF. Not only are MCs producing a series of profibrotic cytokines such as TGF-β, FGF, and PDGF, but they also are considered to be closely related to the formation of microvasculature in OSF by releasing tryptase and chymotrypsin.^110,111^ In addition, the activity of NK cells in OSF patients is significantly lower than that in healthy people.^112^

In the adaptive immune response, CD4^+^ T cells are also important effector cells in OSF pathogenesis.^11^ Myriad CD4^+^ T cell subsets are implicated in OSF development. In a study of 72 patients with OSF and 30 healthy individuals, the researchers identified OSF development associated with enrichment of Th17 cell and IL-17 infiltration occurred in the tissues and PBMCs of patients with OSF.^113^ Compared with healthy individuals, patients with OSF have an increased Th17 cells to regulatory T cells (Tregs) ratio in PBMCs, the former of which is the main producer of IL-17A in fibrotic diseases such as OSF.^113,114^ Other potential profibrotic mechanisms includes the activation of mTOR and MEK induction by amphiregulin derived from Th17 cells, thereby promoting intestinal fibrosis.^115^ The PD-1^+^ Th17 cells contribute to the production of collagen I in lung fibrosis by regulating transcription factor STAT3.^116^ Furthermore, some recent data also indicated that aberrantly activated T cells instigate the transition of fibroblasts into MFBs, which are involved in OSF development. Using scRNA-seq from OSF patients, researchers have revealed that epithelia-mediated T cell activation via CD74/CXCR4 can substantially contribute to the development of OSF.^117^

Other immune cells, such as neutrophils, are one of the main features of early OSF.^118^ However, whether these cells participate in the OSF development remains unclear. Further mechanistic insights and understanding of these immune cells in OSF might facilitate the finding of novel therapeutic targets for patients with OSF.

### Others

#### Oxidative stress

Currently, the detection of oxidative stress and reduced antioxidant defenses has been found in both in the AN chewer and OSF patients. In particular, the activation of reactive oxygen species (ROS) signaling is a primary indicator in the oxidative stress reaction, and the excessive accumulation of ROS will contribute to the initial development of OSF.^119^ Evidence has shown that AN extract can activate p38 MAPK, ERK, and NF-κB signaling to enhance the production of ROS.^82,120^ AN extract can also, through NCOA7(which is an anti-oxidation protective factor) and silencing the expression of miR-155, inhibit ROS levels from fibroblasts of OSF patients.^100,121^ In addition, ROS bind directly to DNA, lipids, and proteins, and leads to DNA base oxidation, generation of lipid peroxidation, and protein carbonylation, respectively, therefore causing oxidative damage of cells. Serum derived from OSF patients showed increased level of 8-OHdG,^122^ malondialdehyde (MDA),^119,123–125^ 4-hydroxynonenal (4-HNE)^126^ and protein carbonyl.^122^ For example, a study including 65 OSF samples showed that the severity of disease was positively correlated with the level of MDA in serum and saliva.^124,125^ Meanwhile, increased ROS related oxidative damage and mitochondrial disruption such as 8-oxoG, 4-HNE, and NOX4 were also verified in OSF tissues.^120,126,127^ Notably, numerous studies in vitro have provided convincing evidence that AN can give rise to oxidative stress, which is mediated through the generation of ROS.^68,100^ First of all, previous studies have confirmed that AN extract can induce oral fibroblasts to secrete a variety of cytokines (such as GRO-α, IL-6 and IL-8) through ROS activation, thus promoting damage of normal epithelium and giving rise to the development of OSF.^120^ Secondly, AN-induced ROS generation activated various pathways leading to aberration of cell cycle and cell viability, EMT, and collagen deposition. AN upregulated the expression of the stress-responsive genes, such as heme oxygenase-1 and ferritin light chain, and induced cell cycle arrest and apoptosis.^82,128,129^ Of note, other evidence also showed that arecoline activates YAP via ROS-induced endoplasmic reticulum stress generation to mediate EndMT, leading to OSF.^17,100^ In addition to excessive ROS generation, reduced antioxidant defense mechanisms contribute to the fibrosis process. Published evidence clearly indicates that the levels of β-carotene and Vitamin E decreased in the plasma derived from OSF patients, and they are known to act in antioxidant roles by quenching lipid peroxidation.^123^ Meanwhile, the reduction of activity and content of antioxidant enzyme, including glutathione (GSH) and superoxide dismutase (SOD) have been also demonstrated in OSF patients.^125,130^ Notably, in vitro studies further indicated that AN extract significantly inhibited GST activity or promoted GSH depletion in human buccal mucosal fibroblasts.^131^ Collectively, oxidative stress, especially the ROS activation in pathogenesis of OSF, is a direction which should be of deep concern and comprehensive exploration.

#### Cellular senescence

Senescent fibroblasts were observed in early OSF, and accumulated with the progress of the disease. Reports in tissues and cultured fibroblasts of OSF have documented accumulation of p16INK4A, the formation of senescence associated heterochromatic foci (SAHF) and high levels of SA-βGal activity in association with OSF.^132^ It is reported that unrepaired DNA double-strand breaks is a major mechanism of senescence. Cultured fibroblasts from OSF tissues showed an increased DNA damaged foci doubly labeled for 53BP1 and γH2A.X accompanied within OSF development.^132^ In particular, the senescence induced by DNA damage was mainly caused by ROS and independent of the shorter telomeres.^133^ ROS-induced senescence has been extensively identified in multiple diseases. A senescent model induced by ionizing radiation in vitro indicated that the expression of p16INK4A and SA-βGal increased in senescent oral fibroblast.^134^ Likewise, the treatment of AN extract also caused cellular senescence phenotypes, as determined by an increased activity of SA-βGal and accumulation of p16INK4A,^135^ which suggested that the development of OSF caused by AN extract may mainly be owed to the role of cellular senescence. On the contrary, a study reported that the fibroblasts of non-diseased (ND) AN users showed no evidence of the increase of senescent cells compared with the healthy controls group.^132^ These data indicated that senescence is associated with intrinsic pathology context in OSF and the mechanism that ultimately leads to OSF development may depend on senescence-related secretory phenotypes (SASPs) derived from senescent cells. Current research revealed senescent oral epithelial cells are involved in OSF development by secreting TGF-β.^136^ Notably, senescent fibroblasts induced by ionizing radiation showed the secretion of TIMP-1 and TIMP-2, and as SASPs, both of them increased in the media of fibroblast cultures from OSF patients where senescent cells are more abundant.^133^ Additionally, most of the OSF biopsies contained significantly higher levels of mRNAs encoding the SASPs components IL-6, IL-8, GRO-α, and IL-1β, but these cytokines have not been verified in the senescent model.^137^ However, current studies have confirmed that SASPs-derived senescent cells are significant in the antifibrosis mechanism, which plays a role by increasing the degradation of collagen. Compared with TIMPs, senescent cells could ameliorate fibrosis by increasing the secretion of MMPs. Reducing the fibroblast population of senescent cells in OSF greatly decreased the levels of MMP-1 and MMP-2 in a conditioned medium indicating that there are both non-fibrotic senescent fibroblasts and collagen-deposited fibroblasts in the cell population.^132^

Overall, the fibrosis-promoting and fibrosis-suppressing functions of senescence seemed to be incompatible, but in fact most senescent cells secrete SASPs that impact fibrosis development, which is a vital mechanism in OSF progression and should be comprehensively explored in the future.

#### Autophagy

Autophagy is a cellular defense mechanism that can improve cell survival by removing useless intracellular organelles or proteins to counteract damage^138^ and is increasingly regarded as an important physiological process of tissue remodeling and fibrosis.^139^ The activation of autophagy promotes α-SMA expression and collagen production in oral mucosa, and it may determine MFBs differentiation.^140^ A previous study has demonstrated the overexpression of microtubule-associated protein 1 light chain 3 (LC3), a marker of autophagy, in OSF tissues. The study in vitro confirmed autophagy in cultured oral fibroblast can be activated by TGF-β, and the inhibition of autophagy significantly reduces the expression of critical fibrogenic gene, Col1A2, and protects against fibrosis induced by TGF-β.^141^ In addition, Zhu et al. revealed that autophagy-related proteins LC3 and P62 are also upregulated in OSF epithelium by immunohistochemistry. The study in vitro further found that the apoptosis of HOKs can be attributed to arecoline-induced autophagy, thereby promoting OSF development.^142^ Similarly, arecoline has been reported to induce autophagy in HUVECs, and activate autophagy while significantly inhibiting angiogenesis and cell viability of endothelial cells. It suggested that the autophagy in endothelial cells may promote OSF by regulating angiogenesis.^143^

#### Microbiota

A few studies hold the idea that microbial flora is also a key trigger that promotes the onset and malignant potential of OSF. According to the linear discriminant analysis, the abundance of Oscillospira and Megamonas was significantly increased in the OSF patient, while Fusobacterium and Rothia was decreased, indicating that it may pose a risk for OSF.^144^ The results of 16S rRNA gene sequencing showed the differences in the bacterial composition at different stages of the progression of AN-associated OSCC.^144^ More importantly, dysbiosis of oral microbiota is a significant risk factor for oral cancer. *Porphyromonas gingivalis* (*P. gingivalis*), *Fusobacterium nucleatum* (*F. nucleatum*), *Treponema denticola* (*T. denticola*), and *Streptococcus anginosus* (*S. anginosus*) have been recognized as being involved in oral carcinogenesis, and several clinical studies detected the relatively significant presence of these bacteria in OSCC sample compared with normal controls.^145^ Notably, previous studies also showed that AN chewing is one of the main reasons for the disturbance of host microbiota.^146^ As a result, it can be concluded that the oral microbiota plays an important role in the onset and transformation of OSF into OSCC, and it has broad prospects for further investigation.

## The clinical evaluation of disease staging in OSF

For the evaluation of OSF progression, a variety of strategies have been proposed in the past few decades. Nevertheless, in clinical practice, finding out the most effective method for assessing oral mucosa impairment in patients remains a tremendous challenge. This is primarily because prognosis and effective therapy are dependent on the assessment of the severity and the extent of OSF patients. Historically, all these parameters were provided by oral mucosa biopsy. Oral mucosa biopsy is among the traditional, effective, and most accurate assessment methods for evaluating oral mucosa histology and the development of OSF. However, in recent years, with the emergence of high-throughput sequencing technology, some biomarkers from the body fluids of OSF patients have also been put forward with the potential for diagnostic value.

### Functional evaluation for OSF development

The estimate of the degree of mouth opening is the most intuitive and convenient way to diagnose OSF in clinical practice. This is because the limited mouth opening is attributed to the submucosal collagen deposition and it is a typical characteristic of OSF.^147^ Of note, the latest guideline was proposed by the Chinese Stomatological Association (CSA) in 2022 and revised the diagnosis and clinical management of OSF. It divided the clinical manifestation of OSF into 4 stages. The characteristic of the first stage is the distance of mouth opening ≥30 mm with partial blanching of oral mucosa, but the texture and elasticity of mucosa have no significant difference. As the lesion develops, the distance of mouth opening is 20–30 mm in the second stage, accompanied by a blanching sheet or stripe in the mucosa. At this point, the texture becomes hard and the elasticity declines. In the third stage, the distance of mouth opening is 10–20 mm, with extensive blanching areas around oral mucosa as well as fibrous bands having been formed. The mucosa shows as a slab or leather change with poor elasticity. In the fourth stage, the distance of mouth opening ≤10 mm, and at this stage, malignant transformation is easily induced, especially into OSCC.

### Oral mucosa biopsy for the evaluation of fibrosis development

In addition to functional evaluation, it is vitally significant to identify that an accurate diagnosis of OSF can only be made when the clinical evaluation and biopsy confer information. A biopsy is the gold standard of diagnosis that is widely used to evaluate and differentiate histological stages of OSF. The pathological change of OSF is mainly collagen deposition in connective tissue, which is characterized by the obvious increase in type I and III collagen. In particular, in histological staging, the number and distribution of fibroblasts, collagen fibers, inflammatory cells, and blood vessels are used to determine OSF grades.^148^ At the earliest stage, there is marked edema of the collagen network, vasodilation, and congestion accompanied by quantities of fibroblasts. The inflammatory cells are mainly composed of polymorphonuclear leukocytes with a small number of eosinophils and the epithelium is essentially normal in this period. In the early stage of OSF, the main characteristics are subepithelial hyalinization, collagen thickening, vasodilation, and congestion, accompanied by a moderate number of fibroblasts. The infiltration of chronic inflammatory cells is dominated by lymphocytes, eosinophils, and a few plasma cells. With the variety of degrees of keratinization, the epithelial nails become shorter. In the middle stage, the epithelium is hyalinized, with thickened collagen bundles and mostly constricted blood vessels. Mature fibroblasts with sparse cytoplasm and spindle-shaped nuclei are obvious. The epithelium is markedly atrophic with a complete loss of reticular nails. Muscle fibers and scattered dense collagen fibers are seen. In some areas, muscle fibers begin to degenerate and show irregular striations. In the advanced stage, extensive fibrosis makes mucosal vessels disappear. Fibroblasts are absent within the area of hyalinization, and occasional elongated cells are seen along the fiber bundles. Epithelial mesh plugs disappear completely, and a few patients show extensive myofiber degeneration.

### Biomarker-based diagnosis of OSF

Although biopsy can effectively assist in clinical diagnosis and observe lesion development of OSF, however, due to the invasive and low acceptance of patients, it is often not an option. The identification of more accessible and less costly biomarkers, such as serum and saliva biomarkers, would increase their use in clinical practice. Various biological biomarkers of OSF have been discovered and characterized in the recent past. The change of trace elements is a typical example. The level of vitamin-c, iron, and zinc was significantly decreased in the serum of OSF patients, accompanied by the increase of copper, and it may indicate serum zinc, copper, and iron levels have potential diagnostic value in OSF.^149–151^ Besides, another study revealed that the serum copper and zinc levels and the Cu/Zn ratio of OSF patients can be regarded as potential markers in the evaluation of the susceptibility towards OSCC.^152^ Bale et al. reported that with the progress of OSF, serum MDA levels were increased while the level of SOD was reduced.^125^ Other potential serum or saliva biomarkers such as lactate dehydrogenase,^153^ and β-carotene,^154^ which may also play an important role in evaluating the development of OSF.

### Non-invasive tools for the identification of OSF

Most strikingly, some novel adjunctive methods are also emerging for the evaluation of OSF. Advances in the understanding of the pattern of epithelial thickness and collagen distribution have enabled the development and validation of numerous computer-assisted techniques for the non-invasive detection of OSF grade. The assessment of collagen by Computer-Aided Diagnosis (CAD) is helpful in identifying OSF grade. For example, Paul et al. reported a new collagen fiber transmission electron microscope image, CAD technology based on Artificial Neural Network (ANN) to identify the progress of OSF.^155^ Additionally, autofluorescence spectroscopy has shown the potential to be a promising technique that can successfully differentiate oral malignancy and OSF.^156–158^ Wang et al. have indicated that the PLS-ANN classification algorithm can effectively differentiate OSF from oral malignant lesions based on autofluorescence spectroscopy at 330-nm excitation.^159^ Vedeswari et al. found that the fluorescence intensity will change due to the fact that there is a difference between the normal tissue and OSF mucosa in the fluorescence emission spectrum after therapy, and it is a useful strategy for the evaluation in the development of OSF.^160^ Kumar et al. further reported a non-invasive strategy by combining in fluorescence spectroscopy with principal component analysis (PCA) and partial least square discriminate analysis (PLS-DA) algorithm to evaluate the development of OSF.^161^ Furthermore, a study reported assessment of the epithelium thickness of OSF by a swept-source optical coherence tomography (OCT) technique.^162^ Rai et al. showed the possibility of FTIR spectroscopy coupled with chemometric techniques to screen OSF patients.^163^ Manjunath et al. reported a novel ultrasonographic technique, which has great superiority for identifying the area and depth of OSF by different submucosal echogenicity between the submucosa and muscle layer.^164^ In addition, micronucleus detection in exfoliated oral epithelial cells is also a non-invasive method used to assess the risk of malignant transformation.^51,165^ As a result, these adjunctive techniques act as a non-invasive strategy that has great potential and should be further investigated in the future.

## Pharmacological treatment of OSF

**Fig. 3:**
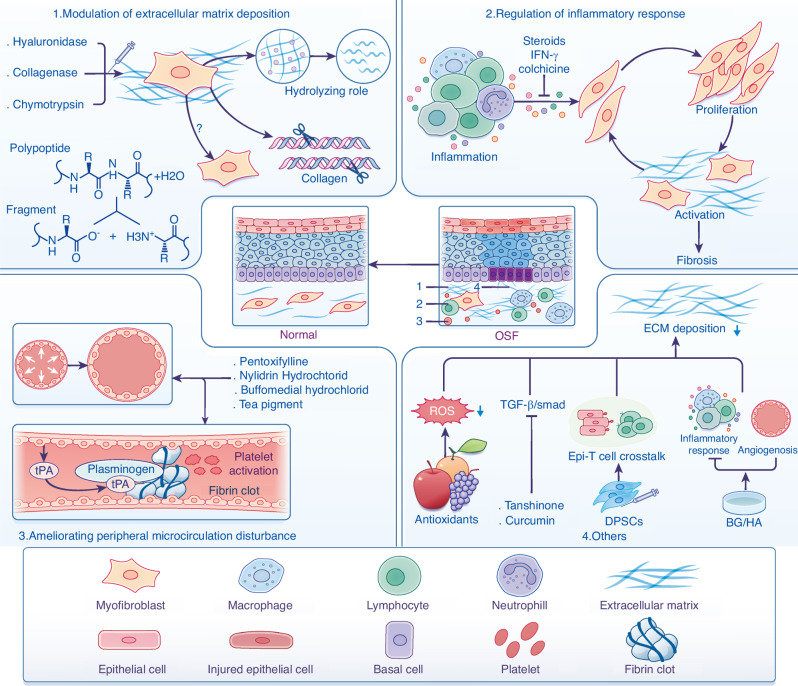
Pharmacological strategies may lead to OSF regression. (1) Modulation of extracellular matrix deposition that control OSF development is an antifibrotic approach: Local injection of hyaluronidase, collagenase, and chymotrypsin is used to reduce the levels of ECM deposition by the hydrolysis of hyaluronic acid, esters, and peptide bonds. (2) Regulation of inflammatory response. Inhibition of inflammation and prevention of activation of MFB by the injection of immunomodulators such as steroids, or anti-fibrotic cytokines. (3) Ameliorating peripheral microcirculation disturbance with established drugs to improve vascular occlusion and fibrinolysis system disorders will attenuate OSF progression, with multiple optimized strategies under development. (4) Other therapeutic strategies are under development to inhibit the collagen aggregation, including antioxidants and natural medicine such as tanshinone and curcumin that act on TGF-β/Smad signaling. BG/HA have been used to inhibit inflammatory responses and promote angiogenesis. DPSCs might have beneficial therapeutic effects via inhibiting crosstalk between epithelial cells and T cells, although further studies are needed

Due to the complexity of pathogenesis, there is no effective modality that provides precise therapy for OSF. The main purpose of treatment is to alleviate the symptoms of patients to the maximum extent, prevent the further progress of the disease and improve the patient’s quality of life. Our present knowledge of the current treatment strategies for OSF are mainly based on etiology, pathogenesis, disease progression and symptoms of patients. The treatment strategies are divided into three categories: medical treatment, functional physical exercise, as well as elective surgery. In particular, medical treatment is the main therapy strategy due to the fact that functional physical exercise and elective surgery are usually suitable for patients with severe OSF. Different drugs that target the potential mechanisms to hinder the development of OSF is necessary. This section briefly recapitulates current drug strategies based on potential mechanisms of OSF, with a focus on possibility that may be considered for future clinical application (Fig. 3).

### Modulation of extracellular matrix deposition

Excessive deposition of ECM in the lamina propria of oral mucosa is an important feature of OSF.^166^ According to previous studies, enzymes such as hyaluronidase, collagenase, and chymotrypsin were applied in the inhibition of ECM deposition and, for many years, it has been regarded as an effective strategy for the treatment of OSF. Hyaluronidase is a naturally occurring enzyme that can hydrolyze hyaluronic acid and reduce collagen formation in extracellular matrix, thus reducing the viscosity of intercellular substances, and hyaluronidase is widely reported to play an important therapeutic role in improve ECM deposition of OSF.^167^ Early evidence demonstrated that OSF patients who experienced local injection of hyaluronidase alone had rapid alleviation in burning sensation.^168^ In addition, collagenase is a type of proteolytic enzyme that was approved by Food and Drug Administration (FDA) and European Medicines Agency (EMA) in therapy of fibrotic diseases such as palmar fibromatosis and Peyronie’s disease.^169,170^ It has a similar effect as hyaluronidase in ameliorating symptoms (e.g., such as trismus, burning sensation) of OSF patients by efficiently cleaving collagen proteins into fragments. Study found that the development of OSF is accompanied by persistent collagen accumulation, and the lack of collagenase significantly contributed to lesion progression. Lin et al. reported that OSF patient, who were injected collagenase significantly improved mouth opening and eating function in a 2-year follow-up study. The study in vivo and histology of biopsy specimens further showed this effect is probably due to the dissolution of submucosal fibrous tissue which led to the increase of vascular circulation and epithelial regeneration.^171^ Of note, chymotrypsin is an endopeptidase that has also been reported in alleviating rational symptoms of OSF patients by hydrolyzing esters and peptide bonds.^167^ However, the effects of these enzymes seem to be transitory due to the labile nature, which will lose their activity completely in a short period of time. Although recently, some studies focus on drug delivery and aim at protecting the activity of enzyme and keeping a sustained controllable release of enzyme for a longer period.^172^ Some studies have also advocated that injection of hyaluronidase combined with corticoids, such as triamcinolone acetonide and dexamethasone, can improve the symptoms of OSF quickly as well as lengthen the effect.^173,174^ The evidence-based medicine shows that these enzymes have insufficient evidence of curative effects, and compelling study of efficiency in therapy was deficient, so they have not been widely popularized in clinical application at present.

Given that MFBs drive collagen formation and ECM deposition, and MFBs are the main source of ECM, it is exciting direction on whether targeted MFB activation will be an effective strategy for OSF treatment in the future.

### Regulation of inflammatory response

OSF has long been considered an outcome of chronic inflammation perpetuating the production of cytokines by tissue-resident cells such as epithelial and mesenchymal cells, as well as infiltrated immune cells such as monocytes, macrophages, and lymphocytes.^118,175^ Currently, therapeutic approaches for OSF have focused on the anti-inflammation role by steroids and corticosteroids. However, these drugs are relatively ineffective in reversing the abnormal deposition of collagen and restoring elasticity of the oral mucosa. They are also usually accompanied by a series of side effects and rare complications such as local swelling and central serous chorioretinopathy.^173,176^ Therefore, it is now commonly accepted that the corticosteroid should be given in combination with other drugs.^167^ CSA recommends the local injection of steroids, especially the triamcinolone acetonide, should be combined with lidocaine or tanshinone, and is effective for alleviating the limitation of mouth opening, oral burning sensation, as well as the area of lesions. This outcome is similar but more effective than that in the clinical studies by Haque et al. who showed that local injection of IFN-γ (50 μg of IFN-γ twice a week for 8 weeks) improved the mouth opening limitation of 29 patients (100%). Of note, the burning sensation and ability to taste also improved, which accounted for 57% and 61%, respectively. The study also showed a reduction of inflammatory cells and levels of cytokines in the lesion sections, and a study in vitro further demonstrated that IFN-γ inhibits collagen synthesis associated with the anti-fibrotic response to arecoline.^177^

Other regimens including tablet colchicine and immunized milk are considered as anti-inflammation agents. Colchicine is a natural alkaloid with anti-inflammatory properties, which inhibits collagen secretion by neutralizing profibrotic cytokines such as IL-4 and TGF-β, thus achieving an anti-fibrotic effect. A clinical comparative study done by Krishnamoorthy et al. on OSF was implemented using 0.05 mg tablets of colchicine twice daily for 12 weeks and found a significant effect in improving symptoms of 50 patients with OSF, including mouth opening limitation and burning sensation (almost 99.8%). The histopathological findings also showed a marked reduction in the inflammatory cells and density of collagen fibrils.^178^ The immune milk produced by immunizing cows with human intestinal bacteria contains anti-inflammatory ingredients, which can inhibit the inflammatory response and regulate the production of cytokines. A clinical study by Tai et al. showed that oral administration of immune milk can improve symptoms and signs in OSF patients. Approximately 69.2% of those who received immune milk (45 mg of immune milk powder twice a day for three months) showed a significant remission in maximum mouth opening with more than 3 mm.^179^ However, the clinical application of immunomodulators is limited to some extent because of their serious side effects and short-term efficacy on OSF.

### Ameliorating peripheral microcirculation disturbance

Some histopathologic studies have demonstrated the phenomenon that there is a progressive loss of vascularity in the local lesion of OSF and is accompanied by hemodynamic disorder. It is suggested that the underlying cause of OSF pathogenesis are due to progressive vascular occlusion and fibrinolysis system disorders. Therefore, some vasodilators such as buflomedial hydrochloride,^180^ nylidrin hydrochloride,^181^ and isoxsuprine^182^ were used to treat OSF by relaxing peripheral blood vessels, restoring blood supply to ischemic tissues, and reducing local tissue hardness. For instance, 10-year clinical research with 150 OSF cases reported that buflomedial hydrochloride combined with vitamin B-complex and topical triamcinolone acetonide 0.1% can effectively improve maximal interincisal distance of OSF patients. Another study also showed that ~62.07% patients with OSF obtained satisfactory improvement in the suppleness of oral mucosa by using nylidrin hydrochloride. In addition, a clinical study in 40 OSF patients revealed that isoxsuprine 10 mg four times per day also showed a desirable effect in improving mouth opening and burning sensation. Of note, a clinical trial by Rajendran et al. showed pentoxifylline, was effective in alleviating burning sensation and mouth opening of OSF patients. Another meta-analysis further deciphered the therapeutic efficacy of pentoxifylline in improving mouth opening limitation over time.^183,184^ Currently, a phase 4 study was carried out to evaluate the efficacy of triamcinolone with pentoxifylline and vitamin E in patients with stage II and III OSF (NCT05660694). Moreover, tea pigment taken orally was also regarded as an effective strategy for OSF treatment due to its ability to reduce blood hyper-viscosity, and improve ischemia, and microcirculation of local mucosa. A previous clinical study in 39 OSF patients demonstrated that oral administration of tea pigment (250 mg twice per day) significantly increased hemorheology in OSF patients and above 58.3% of these patients improved mouth opening by average 7.9 mm.^185^

However, these drugs have certain limitations, and the use of pentoxifylline is usually accompanied by side effects to the gastrointestinal tract and central nervous system, which may limit its clinical popularization. In the long run, other vasodilators are only effective in symptomatic relief, and the long-term effect on OSF is unsatisfactory.

### Others

In addition to the above drugs, some natural compounds with antioxidant effects have also been used in OSF treatment, mainly including lycopene,^186,187^ spirulina,^188^ and aloe.^189,190^ A study on the clinical efficacy of lycopene showed that 21 OSF patients were treated with 16 mg/d lycopene for 2 months, and their mouth opening was obviously improved, with an average of 2.2–4.6 mm.^186^ In addition, some other natural compounds, such as tanshinone and curcumin, which have the characteristics of diverse structures, wide sources, and low toxicity, have attracted much attention in the multi-target therapy of OSF. Tanshinone is an extract from Salvia miltiorrhiza with anti-inflammatory and wound-healing properties, which has been recommended in OSF treatment by guideline of CSA. A study has confirmed the inhibitory effects of tanshinone on OSF development by suppressing arecoline-induced EMT by epigenetically reactivating the p53 pathway.^191^ Tanshinone also effectively inhibited the activation of fibroblasts and collagen aggregation stimulated by arecoline by acting on TGF-β/Smad pathway.^192^ Similarly, curcumin is also a diketone compound extracted from plant roots, which has good anti-inflammatory and anti-tumorigenic properties. A randomized clinical trial of 15 patients with OSF evaluated the therapeutic effect of curcumin, and the results showed that curcumin could effectively improve the burning sensation and mouth opening of OSF patients, without any side effects.^193^

Although above-mentioned clinical studies are certainly limited, available data using AN-induced OSF model suggest that some novel therapies, including sodium hyaluronate/bioglass composite hydrogel (BG/HA) and mesenchymal stem cells (MSCs) are proven to be effective in the treatment of OSF. A typical example that Guo et al. demonstrated the submucosal administration of BG/HA ameliorates the effects of AN by inhibiting the production of pro-inflammatory cytokines, promoting angiogenesis, and reducing collagen deposition.^194^ In addition, recent studies in AN-induced animal models found that the submucosal injection of dental pulp stem cells (DPSCs) can limit the progression of OSF by intervening the crosstalk between epithelial cells and T cells.^117^ Moreover, in vitro study showed that adipose stem cell-derived extracellular vesicles significantly suppressed proliferation, migration, invasion, and expression level of fibrosis marker via the miR-375/FOXF1 axis in fibrotic buccal mucosal fibroblasts.^195^ However, these findings warrant further investigation to explore potential clinical applications. Notably, recent advancements in anti-fibrotic treatments have been directed towards targeting the activation of MFBs, focusing on factors that play in a role in the progression of fibrosis. Pirfenidone (PFD), luspatercept, and AVID-200 are anti-fibrotic drugs which were approved by FDA for pulmonary fibrosis and myelofibrosis, and all of them act as an antagonist for the deposition of ECM by inhibiting the activation of TGF-β.^196^ Tocilizumab, an IL-6 inhibitor, showed effectiveness in the treatment of systemic sclerosis and pulmonary fibrosis,^197,198^ which indicates that the strategies of targeting MFB and immunity have great potential in the treatment of OSF. Above studies indicate that these therapeutic agents have beneficial effects in fibrosis disease, but to date, none of them have been approved for OSF. Further investigation of their role is certainly warranted in OSF.

## Malignant transformation of OSF

The potential of OSF in the malignant transformation was first described by Paymaster in 1956, and it has been regarded as an oral precancerous condition for decades and the WHO redefined OSF as OPMDs in 2005.^199^ However, given that the specific pathogenesis of OSF is not clear at present time, the comprehensive research on the mechanism of its malignant transformation has been greatly hindered. A large number of studies reported that the process of OSF transformation into OSCC is multifactorial, and accompanied by a series of complex biological behavioral changes, which consists of genetic alterations and dynamics of the microenvironments.

### Genetic alteration during OSF carcinogenesis

Multiple studies have reported chromosomal alterations and genetic characteristics of the link between OSF and OSCC. Telomerase reactivation in the chromosome plays a major role in the process of malignant transformation. A typical example is hTERT expression, which shows a strong correlation with telomerase activity. The consistent increase in hTERT levels from normal mucosa to OSF to OSCC tissue samples was also observed by immunohistochemical evaluation.^200^ Moreover, chromosomal alterations, such as copy number variation, have been described in malignant transformation of OSF. The chromosomal 1q21 region harboring S100A14 was found to be deleted during OSF carcinogenesis.^201^ Another cytogenetic study demonstrated the genomic damage caused by AN consumption. Of note, the frequencies of sister chromatid exchanges and chromosome aberrations both increased in peripheral blood lymphocytes of OSF and OSCC patients.^202^ Lin et al. evaluated the polymorphisms of COX-2-765G/G genotype in OSF and OSCC patients by PCR-RFLP methods, and the result revealed COX-2-765C allele vs. -765G/G genotype was a risk factor for malignant transformation of OSF (OR = 3.20, 95%CI = 1.32–8.94).^203^ A previous study evaluated the genetic signatures for carcinogenesis in OSF from 15 blood genomic DNA samples of OSF patients, and the loss of heterozygosity (LOH) loci was observed in over 50% of the OSF tissues. Many of these LOH loci were consistent when compared to OSCC and have been shown to be associated with malignant transformation.^204^ In addition, methylation has also been described in the malignant transformation. Wnt inhibitory factor-1 (WIF1) is regarded as a significant molecule in the progression of OSF malignancy, and the promoter methylation of WIF1 is tumor-specific; it serves as a potential epigenetic biomarker for the OSF carcinogenesis.^205^ Other studies even confirmed noncoding RNAs such as LncADAMTS9-AS2^206^ and circEPSTI1^207^ were also involved in the process of OSF carcinogenesis.

Genomic instability denotes abnormal changes of cell proliferation, apoptosis, as well as cell cycles, thus promoting the process of malignant transformation. Zhou et al. reported that expression of surviving Thr34 phosphorylation, cyclin B1, and p34cdc2 are increased in malignant OSF, and survivin, cyclin B1, and p34cdc2 are key molecules contributing to the malignant transformation of OSF by regulating cell apoptosis and cell cycle.^208,209^ In addition, the Bcl-2 family members, anti-apoptotic Mcl-1 protein,^210^ cyclin D1,^211^ and p63^212,213^ are also involved in the process of malignant transformation.

### Dynamics of the microenvironments during OSF carcinogenesis

As one of the most important features of carcinogenesis, the role of hypoxia and immune balance in shaping the microenvironment of malignant transformation from OSF into OSCC cannot be ignored. The local hypoxia of OSF caused by microvascular lesions may contribute the expression of hypoxia-inducible factor, which is widely regarded as the key molecule to regulate the process of malignant transformation in OSF.^214–216^

Of note, accumulating evidence has also supported the concept of immune surveillance as a key barrier for OSF carcinogenesis. Indeed, the infiltration patterns of dendritic cells (DCs), Th17, and Tregs were demonstrated to be markedly changed in the normal-OSF-malignant transformation. During the malignant transformation of OSF, the increased infiltration of Tregs and decreased expression of DCs are consistent with the formation of a suppressive immune microenvironment. On the one hand, the Tregs expression is statistically higher in OSCC than in OSF, meanwhile Th17/Treg ratio is skewed to favor Tregs. Further analysis showed that Th17/Treg imbalance can be used to indicate a risk factor for malignant transformation of OSF.^113^ On the other hand, the expression of DCs, especially CD1a^+^ and CD207^+^ DCs, is higher in OSCC than OSF.^217^ These results suggest that Tregs and DCs participate in the formation of an immunosuppressive microenvironment and the carcinogenesis of OSF, to a certain extent. Furthermore, previous studies also showed that the MCs may be involved in OSF carcinogenesis.^111^

Most importantly, the abnormal changes of stromal cells, increased matrix stiffness, and senescence also are striking characteristics during the malignant transformation of OSF. The increase of Snail1 and Twist1 were expressed by epithelial cells in OSF during the development of OSF, accompanied by the deletion of E-cadherin, suggesting that OSF has a malignant transformation to OSCC by the EMT process.^218^ Moreover, the abnormal hyperplasia and increased nuclear-cytoplasmic ratio of the basal cell layer are high-risk features for OSF, and these characteristics are closely associated with carcinogenesis.^219^ Another compelling piece of evidence is the basal stem cell in OSF with abnormal self-renewal capacity, which contributes to the malignant transformation of OSF.^220^ In addition, the progression of OSF is accompanied by the increase of matrix hardness, which can promote the migration phenotype by mediating the nuclear transfer of YAP/TAZ.^221^ The increased matrix stiffness was caused by excessive deposition of ECM, which is closely associated with activation of MFBs. Punnya et al. confirmed that as the OSF was malignantly transformed, it was accompanied by epithelial cell dysplasia, as well as the increase of α-SMA^+^ MFBs, thus enhancing local tissue stiffness.^59,222^ Furthermore, the senescence of fibroblasts and epithelial cells will lead to malignant transformation by secreting SASPs.^137,223^ Altogether, stromal cells, matrix stiffness, and senescence have been shown to play a role in the crosstalk between OSF progression and oncogene activation.

## Perspectives and conclusions

At present time, it is undeniable that OSF is a disease with a malignant tendency, and the specific pathogenesis of its occurrence and malignant transformation is not clear yet. In spite of enormous efforts, non-invasive, effective diagnostic approaches, precise therapy strategies, and available preventive methods are the ultimate aims to vanquish this disease. In addition, a favorable animal model is an urgent requirement for the comprehensive study of OSF. Existing in vivo models of OSF are mainly mice or rats induced by arecoline or bleomycin.^38,194,224,225^ Both of them only replicate similar pathological conditions, which may not accurately simulate the pathogenesis of OSF, leading to the research on the treatment of OSF being greatly limited. Thus, the research focus should be on the construction of in vivo models, and on this basis, research in the field of OSF administration should be carried out.^226^ Unfortunately, some intractable questions and challenges remain.

First, most studies reported that organ fibrosis (heart, liver, kidney, lung, etc.) is usually mediated by the type II immune response, which is a chronic inflammatory response, but it has not been identified in OSF. The immunological research of OSF is limited to the phenotypic changes of immune cells and related cytokines, but the key immune cells or cytokines have not been figured out in vivo. Secondly, with regard to the pathogenesis of OSF, the EMT of epithelial cells has currently received a lot of attention. However, the importance of basal stem cells is usually ignored, and the integrity of basal stem cells is crucial for epithelial homeostasis. Most importantly, a recent study has reported reduced stem cell activity in OSF.^220^ As a result, the interaction between basal cells and submucosal cells should be considered for further investigation. Third, the role of human microbiota in organ fibrosis is one of the hot research topics currently. Dysbiosis of oral microbiota, which could damage the epithelial barrier, activate immune responses, and stimulate submucosal cells, may be related to the progression and malignant transformation of OSF. According to the present viewpoint, microbiota biogeography plays a vital role in disease progression.^227^ The field of oral microbiota may recognize the importance of spatially resolved information in understanding the complexity of the oral ecosystem and its potential role in mucosal diseases such as OSF. However, it is challenging to prove the causal relationship between microbiota and oral mucosal diseases, and most findings only exhibit correlation rather than causal evidence so far. Whether the occurrence and development of OSF can be attributed to a single pathogen remains controversial, and it’s even harder to reach a consensus on major pathogenic components of the complex oral microbiome. There are still many questions about the interaction between the microbiota and host cells in a certain ecological niche of the oral mucosa that have yet to be unraveled. Finally, although we have made impressive progress in understanding the pathogenesis of OSF in the past few years, we still need to overcome multiple challenges to turn this information into effective anti-fibrosis therapies. At present, there have been pharmacological therapies aimed at improving local blood circulation and targeting inflammatory response. Surprisingly, few of the medical therapies currently proposed are directed at MFBs, which is the core of the pathogenesis of OSF. Therefore, the new direction aims to explore strategies of drugs that target MFBs of OSF.

Taken together, chewing AN is the main factor for OSF. Although it is known that a variety of pathogeneses of OSF exists, events that contribute to the onset and development of OSF are still not fully understood. Deciphering the biology that underlies these events will enable the devising and testing of novel and effective antifibrotic therapies. Additionally, there is also a demand for improving diagnostic approaches that can early, accurately, and noninvasively evaluate fibrosis, which will allow for the prevention of malignant transformation of OSF and improve the quality of individuals’ life.
